# TSGene 2.0: an updated literature-based knowledgebase for tumor suppressor genes

**DOI:** 10.1093/nar/gkv1268

**Published:** 2015-11-20

**Authors:** Min Zhao, Pora Kim, Ramkrishna Mitra, Junfei Zhao, Zhongming Zhao

**Affiliations:** 1 School of Engineering, Faculty of Science, Health, Education and Engineering, University of the Sunshine Coast, Maroochydore DC, Queensland 4558, Australia; 2 Department of Biomedical Informatics, Vanderbilt University School of Medicine, Nashville, TN 37232, USA; 3 Department of Cancer Biology, Vanderbilt University School of Medicine, Nashville, TN 37232, USA; 4 Department of Psychiatry, Vanderbilt University School of Medicine, Nashville, TN 37212, USA; 5 School of Biomedical Informatics, The University of Texas Health Science Center at Houston, Houston, TX 77030, USA

## Abstract

Tumor suppressor genes (TSGs) are a major type of gatekeeper genes in the cell growth. A knowledgebase with the systematic collection and curation of TSGs in multiple cancer types is critically important for further studying their biological functions as well as for developing therapeutic strategies. Since its development in 2012, the Tumor Suppressor Gene database (TSGene), has become a popular resource in the cancer research community. Here, we reported the TSGene version 2.0, which has substantial updates of contents (e.g. up-to-date literature and pan-cancer genomic data collection and curation), data types (noncoding RNAs and protein-coding genes) and content accessibility. Specifically, the current TSGene 2.0 contains 1217 human TSGs (1018 protein-coding and 199 non-coding genes) curated from over 9000 articles. Additionally, TSGene 2.0 provides thousands of expression and mutation patterns derived from pan-cancer data of The Cancer Genome Atlas. A new web interface is available at http://bioinfo.mc.vanderbilt.edu/TSGene/. Systematic analyses of 199 non-coding TSGs provide numerous cancer-specific non-coding mutational events for further screening and clinical use. Intriguingly, we identified 49 protein-coding TSGs that were consistently down-regulated in 11 cancer types. In summary, TSGene 2.0, which is the only available database for TSGs, provides the most updated TSGs and their features in pan-cancer.

## INTRODUCTION

Cancer is a large family of diseases that cause millions of death worldwide every year (1). It is characterized by the abnormal cell growth with a potential to spread through the body. It often arises from two types of genetic alterations related to the cell proliferation, differentiation, apoptosis and cell-to-cell communication (2,3): the loss-of-function of tumor suppressor genes (TSGs) and the gain-of-function of oncogenes (OCGs). The inactivation or reduced function of protein-coding TSGs can be induced in many ways including promoter methylation changes (4), copy number alterations (5), deregulated mRNA expression due to microRNA (miRNA) activities (6) and competing endogenous long non-coding RNAs (lncRNAs) (7). In general, TSGs play key roles in the cell cycle checkpoints and in maintaining genomic stability. Defective TSGs often allow uncontrolled cell growth without normal DNA repair, apoptosis and normal metabolic regulation (8). Accumulating lines of evidence have shown that non-protein coding RNAs, such as miRNAs (9–11) and lncRNAs (12), can act as TSGs to initiate and promote cancer development.

To provide a comprehensive TSG resource for the cancer research community, we developed the Tumor Suppressor Gene database (TSGene 1.0) in 2012 (13), and have been continuously maintaining it since then. TSGene 1.0 is the only active data resource specifically designed for TSGs. It has received 82573 web hits based on daily unique internet protocol addresses. Since its release, TSGene database has become a popular resource, enjoying wide use for testing drug resistance (14), studying HIV integration of cancer-related genes (15,16), exploring phosphorylation regulatory networks in cancer cells (17), identifying cancer-associated transcript fusions (18), uncovering intronic enhancers through loss of methylation (19) and designing genome-scale CRISPR-based gene repression (20). Moreover, TSGene 1.0 has been frequently used as a special gene list in the systems biology-based studies for the cancer genomic data (21–24).

In the past several years, we have witnessed the unprecedented growth of cancer genomic data, such as those from the Cancer Genome Project (CGP), The Cancer Genome Atlas (TCGA) and the International Cancer Genome Consortium (ICGC). Furthermore, many more TSGs have been reported including non-protein coding genes (miRNAs and lncRNAs). Accordingly, there is a strong need to characterize the tumor suppressor gene landscape at the genome, epigenome, transcriptome and proteome levels, across all types of cancer. We have addressed this need with substantial updates to TSGene 2.0. Our changes include more extensive literature curation, data integration and annotation, and a user-friendly web interface.

As the only literature-based database dedicated to TSGs, TSGene 2.0 provides not only a comprehensive resource for the cancer research community, but also a classified TSG catalog for advanced integrative analyses across multiple cancers. For example, as described in this paper, we observed that two lncRNA TSGs, *DLEU1* and *DLEU2*, are not only highly mutated in multiple prostate cancers, but also in cancers of the bladder and ovaries. Our analyses of 24 highly confident miRNA TSGs revealed that they were associated with cancer-specific signaling pathways in multiple cancers. Furthermore, we pinpointed a testable prevalent deletion of *has-miR-31* in high-grade glioblastoma (GBM). We also used a pan-cancer expression data analysis method and found that the TGF-beta signaling pathway is dominated by TSGs that are consistently down-regulated in tumor samples. These cancer genomics-based integrative analyses can provide complementary evidence of novel functions of known TSGs in the new cancer types with potential lethal effects that otherwise might have been overlooked in the analysis of individual cancers. TSGene 2.0's new web interface has more user-friendly features for browsing the relevant information and querying the functionalities of TSGs. The web server is available at http://bioinfo.mc.vanderbilt.edu/TSGene/.

## DATA COLLECTION AND DISCUSSION

### Curation of the known and conflicting TSGs in literature

To maintain consistency, we duplicated the literature querying strategy used for TSGene 1.0 on the PubMed and GeneRif (Gene Reference Into Function). The most recent systematic PubMed search was conducted on 25 April 2015 using the term: ‘tumor suppressor’ [Title] NOT (P53 [Title] OR TP53 [Title]). To avoid false results, we searched for matches in titles only. The search returned 6178 PubMed abstracts. On the same day, we also extracted 5454 additional short statements associated with 3719 PubMed abstracts from GeneRif database (25), using the term ‘tumor suppressor.’ For new gene type, long non-coding RNAs, we performed extensive PubMed searches separately by using the expression: ‘long non-coding RNA’ and ‘tumor suppressor.’ This search returned 357 references for further curation. After removing the 5795 references that have been analyzed in our previous curation, we kept ≥3000 references for the manual check. Following our previous reference curation processes, we first downloaded all the abstracts from the PubMed and grouped them according to semantic similarity. Next, we extracted the sentences containing the keyword ‘tumor suppressor’ and manually extracted gene names and translated them to the official gene symbols from Entrez Gene database.

In this round, we were more cautious for the TSGs with potential oncogenic roles. TSGs may also play different roles in different cancers or at different stages of disease. For example, histone deacetylase 1 gene (*HDAC1*) has been reported as a TSG during the cancer initiation, but as an oncogene during the tumor maintenance process (26). Other TSGs may have oncogenic roles in different cancer grades. For example, *RASSF1* is a TSG mainly reported in neuroendocrine tumors of the lung. One of its isoform acts as an oncogene in some high-grade lung tumors (27). The Notch signaling pathway has been identified as oncogene in the hematopoietic cancers (28). However, accumulating lines of evidence suggest that pathway members have growth-suppressive roles in some hematopoietic cells, in skin, pancreatic epithelium and hepatocytes (28). *SIRT1* can negatively regulate the TGF-beta signaling pathway and enhance tumorigenesis, but it also interacts with promyelocytic leukaemia (PML) protein to stabilize *TP53* and induce cell senescence (29). *WT1*, a well-studied tumor suppressor has dual roles in cancer progression depending on the presence or absence of regulatory protein partners (30). A few well-studied oncogenes, such as *MYC*, have weak evidence for tumor suppression (31). We did not include them in TSGene database to avoid potential misuse.

To create an overview of TSGs that have also been reported to act as oncogenes, we compiled a gene list of 320 protein-coding oncogenes. Our information sources included a classical review of cancer genes (32), a research article for oncogenic miRNAs (33), the UniProtKB keyword ‘proto-oncogene’ and 17 TSGs with dual roles from our literature curation (Supplementary Table S1). As the result, we obtained a list of 73 TSGs with potential oncogenic roles (including 54 protein-coding TSGs and 19 miRNA TSGs) (Supplementary Table S2). This list of 73 genes is also available through TSGene 2.0. We will update it frequently.

In summary, we compiled 1217 human TSGs, including 1018 protein-coding and 199 non-coding genes, from 3354 PubMed abstracts with confirmed literature evidence. We stored all the curated TSGs and relevant annotations in a MySQL relational database. A dynamic web interface was implemented by Perl CGI and JavaScript for data browsing and querying.

### Representative entries in the TSGene 2.0

Supplementary Figure S1 shows a compilation of information provided by TSGene 2.0. Annotations for each gene can be obtained by clicking the links at the top left (General information, Expression, etc.). *General information* (Supplementary Figure S1A) displays the gene name, pathway, disease, nucleotide sequence and protein sequence. Supplementary Figure S1B shows highlighted summaries from supporting literature and other data sources. The *Expression* page has differential gene expression plots in 11 cancer types; they are provided in a box view with cancer type and average expression score (Supplementary Figure S1C). Taking *BRCA1* as an example, the expression plot shows relatively higher expression in ten cancers compared to matched normal tissues (all adjusted *P*-values <0.05; Student's *t*-test; Supplementary Figure S1C). To help users obtain results of statistical analysis, we present all statistical *P*-values for each TSG on the Expression page. Experimentally verified miRNA targets and predicted upstream transcription factors for each TSG are provided on the *Regulation* page (Supplementary Figure S1D). Somatic mutational annotations for each TSG from the COSMIC database are shown on the *Variant* page. The Lollipop plots in Supplementary Figure S1E, the pie charts in Supplementary Figure S1F and the bar plots in Supplementary Figure S1G summarize somatic mutational features on protein domains, cancer types and ratios of loss-of-function over missense mutations, respectively. We also used a copy number variation plot to present the copy number profile for each TSG. Finally, the *Interaction* page shows physical interaction information. It is supported by integrated high-throughput experiments, metabolic and signaling interactions, and helps users explore partners interacting with each TSG (34).

### Text querying, sequence searching and data browsing

TSGene 2.0 provides text-based query and BLAST search functions (Supplementary Figure S2). A quick text search box on the top right of each page is used to query by gene symbol and Entrez gene ID. The advanced search page provides access to various TSG annotations, including gene symbol, Entrez gene ID, genomic location, disease and pathway (Supplementary Figure S2A). Users can also retrieve any specific TSG set by keyword matching. Sophisticated logical operators provide a window for the user to retrieve somatic mutation information on customized mutations, tumor types and histology types. Moreover, annotations on interactors, transcription factors and regulatory information are also searchable (Supplementary Figure S2A). An updated BLAST interface is provided for the newly collected TSGs in our TSGene 2.0 database (Supplementary Figure S2B).

All TSGs and related information are available for download for analysis or large-scale project design. The data browsing functions in TSGene 2.0 are improved, and provide quick access to a specific set of TSGs. Users can browse TSGs using a KEGG pathway, chromosome, gene type, cancer type, summarized expression and mutational feature (Supplementary Figure S2C–F).

### Functional annotations of 19 lncRNA TSGs

**Figure 1.**
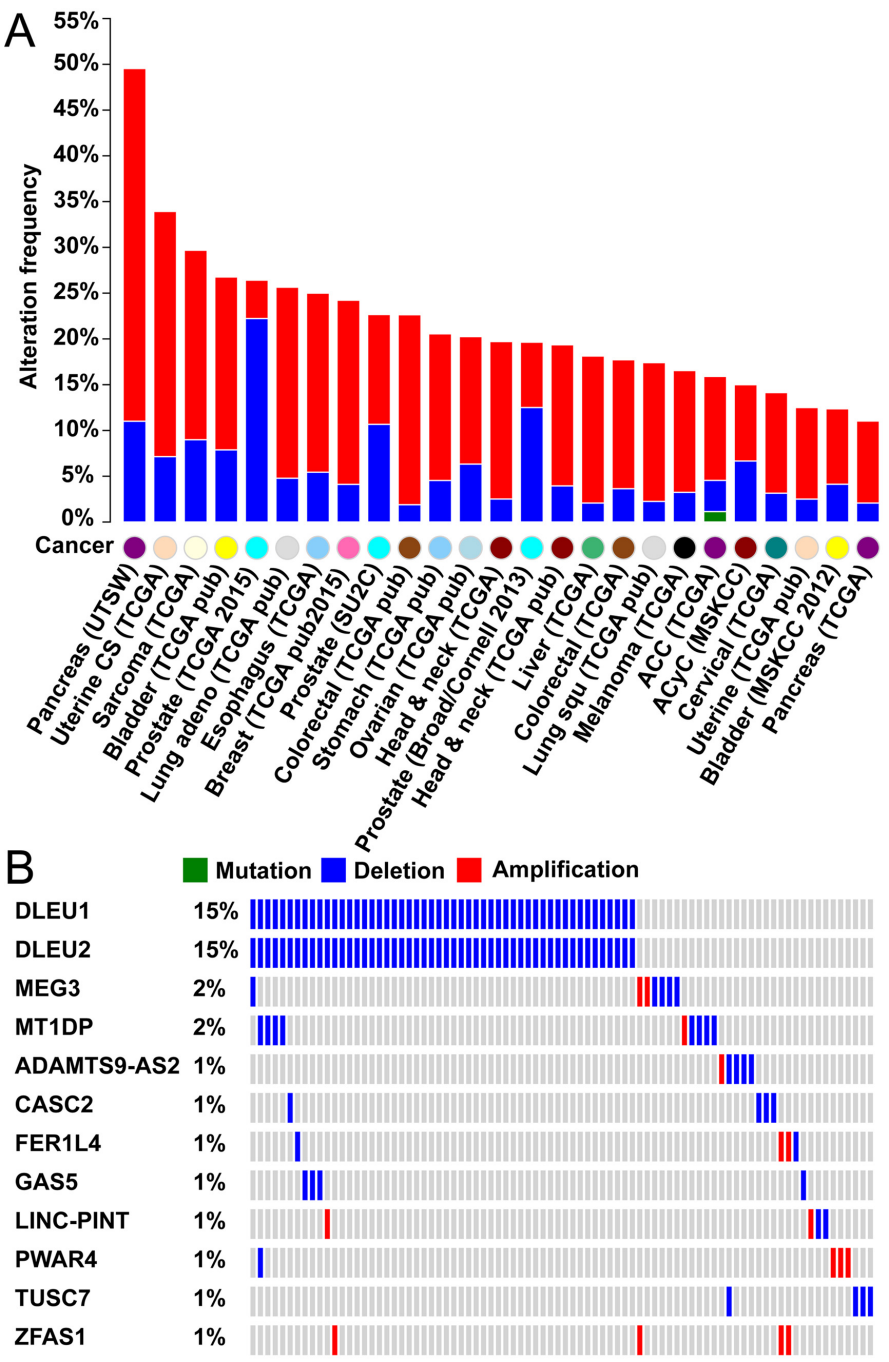
Alteration landscape of the 19 long noncoding RNA (lncRNA) TSGs in pan-cancer. (**A**) Alteration profiles of 19 lncRNA TSGs in 25 major cancer types with alteration frequency ≥10% in each cancer type. (**B**) Sample-based distribution of alterations in 19 lncRNA TSGs in TCGA prostate cancer data. CNA: copy number alteration. Multiple alterations: more than one type of mutations. All the mutational analyses were conducted based on the cBIO portal data (45).

TSGene 2.0 contains information on 19 lncRNAs with the tumor suppressive features (Table 1). To explore mutational features of these 19 lncRNA TSGs, we mapped their genomic coordinates into TCGA's publicly available genomic alteration data (Figure 1A). We found genetic alterations related to them in 54 cancer types. Alteration frequencies were ≥10% in 25 cancer types. Prostate cancer was the most prevalent, with >22.6% of TCGA prostate tumor samples harboring copy number losses (Figure 1A). Using the TCGA prostate data, we plotted the alteration profiles of these lncRNAs. The most frequently altered lncRNAs were *DLEU1* and *DLEU2*, followed by *MEG3* and *MT1DP*, both of which had the alteration frequency >2% in 313 TCGA prostate cancer patients (Figure 1B). This mutational pattern in prostate cancer may provide information about the complementary functions in their tumor suppressive roles of these genes.

**Table 1.** Nineteen long non-coding RNAs with tumor suppressor roles

Gene symbol	Cancer tissue / cell line	References
*ADAMTS9-AS2*	Glioma	(46)
*CASC2*	Colorectal, endometrial, gliomas	(47,48)
*DLEU1*	Leukemia	(49)
*DLEU2*	Leukemia	(49,50)
*FER1L4*	Gastric	(51)
*GAS5*	Breast	(52)
*H19*	Breast, hepatocellular, embryonal tumor cell line	(53)
*LINC-PINT*	N/A	(54)
*LOC401317*	Nasopharyngeal	(55)
*MEG3*	Meningioma, hepatocellular, leukemia, pituitary tumor	(12)
*MT1DP*	Hepatocellular	(56)
*PTCSC3*	Thyroid	(57)
*PTENP1*	Colon, hepatocellular, prostate	(12,58)
*PWAR4*	Breast, endometrial	(59)
*TDRG1*	Testicular	(60)
*TP53COR1*	Lymphoma	(12,61)
*TUSC7*	Colon	(62,63)
*XIST*	Hematologic	(64)
*ZFAS1*	Breast	(65)

Considering their high alteration frequency, *DLEU1* and *DLEU2* may be the candidate TSGs for further experimental evaluation in prostate cancer. To predict their possible biological functions, we examined their co-expressed protein-coding genes using the TCGA prostate RNAseq quantitative scores with both mRNA and lncRNA expression data (Supplementary Table S3). For *DLEU1*, only one gene, *TRIM13*, had a Pearson's correlation coefficient (PCC) ≥ 0.5. *TRIM13* encodes an E3 ubiquitin-protein ligase with three zinc-binding domains and a GTPase activating protein domain in the amino acid terminus (35). It is involved in the formation of intracellular vesicles transporting and phospholipase D activation (35). Different from *DLEU1*, *DLEU2* had 17 protein-coding genes with the PCC values ≥ 0.5, including a tumor suppressor gene (*E2F2*). Functional enrichment analysis of these 17 genes found that they were mainly associated with the cancer pathways or cancer-related gene interactions (Supplementary Table S4). We also observed a number of amplifications for the collected 19 lncRNAs in many cancers (Figure 1A), which might warrant further experimental validation for their potential oncogenic roles in those cancers.

### Biological features of the 179 miRNA TSGs in humans

In recent years, an increasing number of miRNAs have been reported as functioning as tumor suppressors. We have collected 179 tumor suppressor miRNAs; they have important roles mainly in suppressing oncogenes, thus, inhibiting tumor growth. To assess their relationship to protein-coding TSGs, we picked 13 well-studied TSGs, each having the evidence supported by at least 30 publications (Supplementary Table S5). We used a gene ranking tool, ToppGene (36), to prioritize the tumor suppressor miRNAs and found 24 miRNAs that were significantly associated with the 13 TSGs (Supplementary Table S5, ranking *P*-values < 0.05). To further explore the functional distribution of the top ranked miRNAs, we performed a target-based functional enrichment analysis using DIANA-miRPath (37) (Table 2). Not surprisingly, the majority of the enriched pathways were related to the key cancer pathways, such as the cell cycle and tumorigenesis. By incorporating the somatic single nucleotide variant (SNV) and copy number variation (CNV) data in these 24 miRNAs, we created a mutational landscape of these critical tumor suppressor miRNAs in pan-cancer manner. They were mutated in 486 cell lines (55.20%) in the Cancer Cell Line Encyclopedia (CCLE) data set. Because miRNAs are relatively short in terms of nucleotide sequence, the high mutational rates observed in the 24 key miRNAs may reflect their critical and complex roles in cancer cells.

**Table 2.** Top 20 KEGG pathways enriched with the 24 microRNA TSGs

KEGG pathway	Adjusted *P*-value*	# genes	# microRNAs
Viral carcinogenesis	5.65E-27	43	14
Cell cycle	6.81E-24	33	13
Pathways in cancer	1.51E-20	48	14
Small cell lung cancer	7.98E-17	21	13
Hepatitis B	7.98E-17	28	14
Chronic myeloid leukemia	1.73E-15	19	13
Colorectal cancer	5.48E-14	16	12
Prostate cancer	5.48E-14	20	12
Endometrial cancer	2.70E-10	12	11
Pancreatic cancer	9.02E-10	16	10
Transcriptional misregulation in cancer	1.21E-09	29	11
HTLV-I infection	1.25E-09	33	13
Bladder cancer	6.19E-09	11	9
p53 signaling pathway	7.70E-08	14	11
PI3K-Akt signaling pathway	4.48E-07	36	14
Non-small cell lung cancer	8.52E-07	11	11
Measles	1.30E-06	20	10
Glioma	3.51E-06	12	13

*P-values were calculated by hypergeometric tests followed by the Benjamini-Hochberg multiple testing correction (66).

**Figure 2.**
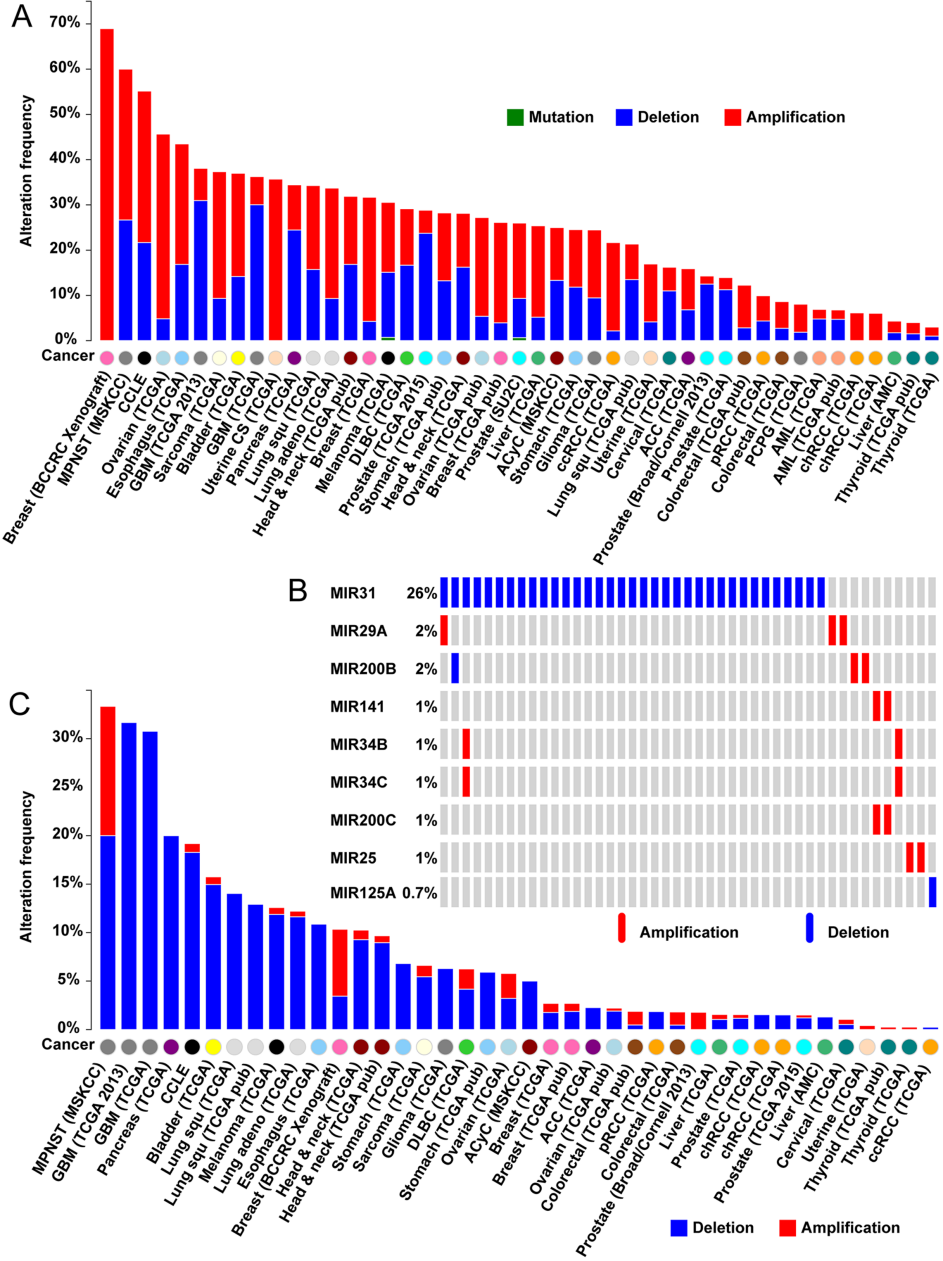
Mutational landscape of the 24 top-ranked microRNA (miRNA) TSGs in pan-cancer. (**A**) Somatic mutational patterns of 24 top-ranked miRNA TSGs in multiple cancer types. (**B**) Sample-based copy number alteration covering 9 miRNA TSGs in TCGA high-grade glioblastoma. (**C**) Global somatic mutations of *has-miR-31* in multiple cancer types. All the mutational analyses were conducted based on the cBIO portal data (45).

As shown in Figure 2A, more patients with ovarian and esophageal cancers carried any type of alteration of the 24 miRNAs, and deletion events were most frequent in glioblastoma multiforme (GBM). In total, there were 10 mutated miRNAs in the TCGA GBM cohort (Figure 2B). This observation is consistent with our previous pathway analysis results (Table 2) showing that the 24 miRNAs were enriched in glioma (corrected *P*-value = 3.51E-06). Deletion of *hsa-miR-31* was detected in 26% of GBM samples (Figure 2B). According to the record in our TSGene database, *Has-miR-31* has been characterized as an miRNA TSG in breast cancer (38). However, systematic study of its role in human cancers is lacking. Given that TSGs mainly operate by the ‘loss-of-function’ model, we further explored the deletion frequency of *has-miR-31* in each cancer type. Figure 2C shows that it was highly deleted in both GBM and pancreatic adenocarcinoma (20.0% of the cohort). It is reported that *has-miR-31* functions to inhibit GBM migration and invasion (39). Its influence on the risk of GBM was also confirmed by the survival analysis in a mouse model (39). Further screening of patients with *has-miR-31* deletions may find more clues about its roles in cancer metastasis and progression.

### protein-coding TSGs consistently down-regulated in pan-cancer

**Figure 3.**
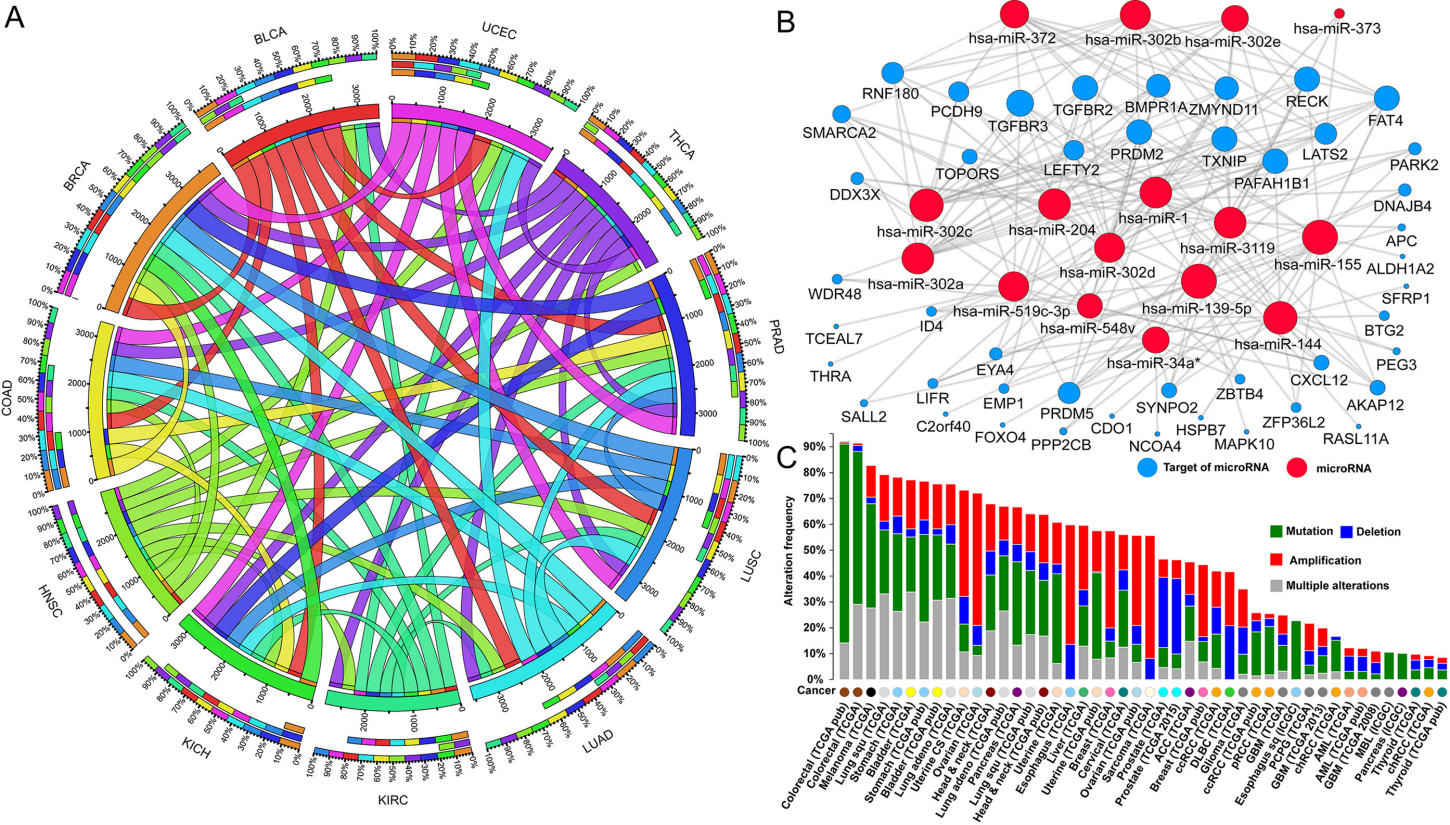
Biological features of 49 down-regulated TSGs in multiple cancer types. (**A**) Shared down-regulated TSGs in 11 major cancers. Eleven colors represent 11 cancer types, respectively. Lengths of the circularly arranged segments are proportional to the total number of TSGs in the 11 cancers. The three outer rings are stacked bar plots, representing relative overlap of other cancer TSGs to the cancer totals. Ribbons connecting different segments represent the number of shared TSGs between cancer types. (**B**) A microRNA regulatory network enriched with the 49 down-regulated TSGs. The network contains 16 microRNAs (red) and 45 down-regulated TSGs (blue) targeted by these connected microRNAs. (C) Global view of the somatic mutations in the 49 down-regulated TSGs in pan-cancer.

Based on the pan-cancer expression profiles in 11 cancers from TCGA, we surveyed gene expression changes for all TSGs by comparing expression in the tumor and control samples (Supplementary Table S6). We identified 8351 differentially down-regulated events among 1027 TSGs in 11 cancers (Student's *t*-test, all adjusted *P*-values <0.01). A total of 1022 TSGs were down-regulated in at least 2 cancer types (Figure 3A), suggesting that they may be consistently down-regulated in multiple cancer types. Importantly, we found 49 TSGs whose expression was consistently decreased in all the 11 cancer types (Supplementary Table S7). Pathway enrichment analysis indicated that these 49 genes were enriched in the TGF-beta signaling pathway (adjusted *P*-value = 2.03E-4) (Supplementary Table S8). The Gene Ontology (GO) analysis revealed that the genes are mainly involved in the regulation of cell proliferation pathways (Supplementary Table S8). Interestingly, they were enriched in the experimentally verified targets of 16 miRNAs (Figure 3B). Three of these microRNAs are well-characterized oncogenes (*has-miR-21*, *has-miR-372* and *has-miR-373*) (40). This finding may indicate a similar competitive regulatory pattern between oncogenic miRNAs and TSGs (22).

Although these 49 genes were down-regulated in tumor samples, their mutational features have not been systematically explored. To this end, we plotted all the somatic alterations, including single nucleotide variants and gene copy number alterations, using all publicly available TCGA cancer data (Figure 3C). Remarkably, the 49 genes were highly mutated in 24 cancer data sets, affecting over 50% of patients in each cancer cohort. The cancer in which these genes were most frequently mutated was colorectal, with 92% of tumor samples having at least one mutational event in these 49 genes (Figure 3C). Further examination of the sample-based mutational patterns in this cancer revealed that the *APC* genes were most frequently mutated among the 49 genes, occurring in 78% of the tumor samples (Supplementary Figure S3). Additionally, the mutational frequency was >10% in 8 other TSGs in colorectal cancer: *FAT4* (19%), *TOPORS* (15%), *PPP2CB* (13%), *RASL11A* (13%), *PCDH9* (12%), *PRDM2* (12%), *LIFR* (11%) and *TGFBR2* (11%). Recently, *FAT4* was reported as a recurrently mutated driver gene in the colorectal cancer (41). However, the lack of evidence for other TSGs like *TOPORS* in the colorectal cancer suggests that future studies are needed.

## SUMMARY AND LIMITATIONS

We have updated our TSGene database to version 2.0, which catalogs 1217 human TSGs curated from thousands of publications. This updated database has an additional list of 572 TSGs that were manually curated from the newly published studies. Its comprehensive annotations, including the pan-cancer gene expression and mutational profiles, provide useful resources for further exploration of the biological functions of TSGs and for cross-cancer comparison of TSGs. In addition, the massive precomputed results and graphic presentations in TSGene 2.0 will be helpful for the cancer-specific TSG identification and subsequent analyses.

Although we performed extensive literature searching and curation, it is important to acknowledge the difficulty of performing an error-free search. For example, our strict search strategy, which matched the keyword to the reference title, caused us to miss some newly reported, but not widely accepted TSGs. We overcame this shortcoming in this update by performing an extensive literature search. In our reference curation, we collected a number of TSGs with potential oncogenic roles. Based on oncogenes collected from public resources, we pinpointed 73 TSGs with such roles. We would suggest that users use the TUSON score (42) (integrated in our database), which is a predictive score of TSGs and oncogenes based on their mutation profile patterns. Following the approach of TUSON, we compared the mutation ratio between the loss-of-function mutations and missense mutations based on the COSMIC annotations to provide additional information for the user. We defined loss-of-function mutations by extracting the following 7 mutation types from COSMIC: (i) frameshift deletion, (ii) whole gene deletion, (iii) complex in-frame deletion, (iv) in-frame deletion, (v) frameshift insertion, (vi) frameshift and (vii) nonsense substitution. Next, we counted the number of missense mutations for each TSG and calculated the ratio of the number of loss-of-function mutations to the number of missense mutations. The results are shown by color bars in our web page: blue denotes the relative abundance of loss-of-function mutations and green denotes the relative abundance of missense mutations. Although the majority of TSGs, such as *TP53*, had fewer loss-of-function mutations than missense mutations (709 TSGs with ratios between 0 and 0.2), some well-known TSGs like *RB1* and *APC* had more loss-of-function than missense mutations (i.e. ratio >0.5). *RB1's* loss-of-function mutation ratio was 0.58. *APC*, had the highest ratio (0.84). From these results, we may infer the relative loss-of-functional impact. Such a compilation of diverse data sets or information in TSGene 2.0 enables the researchers to assess different lines of evidence or features for their specific projects.

There are some limitations in our annotations. For example, we only provide the longest representative DNA and protein sequences for each TSG, and do not cover all potential isoform sequences. We added a note to this effect on the page for sequence information, so that users are aware that TSGene 2.0 provides only the longest representative sequences. These sequences are useful for human research, but some homologous sequences may not be found in other species if a user performs a BLAST-based sequence similarity search. For protein-coding genes, it may be feasible to collect homologous sequences from widely-used databases like HomoloGene (43). However, there is still a lack of high-quality homologous data for non-coding genes, particularly for long non-coding RNAs.

We will continue to update the TSGene database as new information appears, especially data related to noncoding RNA, proteomics and metabolomics data. TSGs are involved in multiple steps in cancer progression, including initiation, progression and metastasis. For example, a recently described cancer metastasis suppressor database (44) has many overlapping between metastasis suppressors and TSGs. Therefore, we also plan to curate more specific information for each TSG, such as tumor type(s) and subtype(s), as well as data about cancer initiation, progression and metastasis. In addition, we will further integrate high-throughput genomics data like epigenetic changes for each TSG. Finally, we plan to add more useful annotations and add homologous genes in other model species. Our goal is to provide a continuously updated, high quality and content-rich literature-based TSG database to facilitate the TSG studies.
